# Optimal Predator Risk Assessment by the Sonar-Jamming Arctiine Moth *Bertholdia trigona*

**DOI:** 10.1371/journal.pone.0063609

**Published:** 2013-05-06

**Authors:** Aaron J. Corcoran, Ryan D. Wagner, William E. Conner

**Affiliations:** Wake Forest University, Department of Biology, Winston-Salem, North Carolina, United States of America; University of Southern Denmark, Denmark

## Abstract

Nearly all animals face a tradeoff between seeking food and mates and avoiding predation. Optimal escape theory holds that an animal confronted with a predator should only flee when benefits of flight (increased survival) outweigh the costs (energetic costs, lost foraging time, etc.). We propose a model for prey risk assessment based on the predator's stage of attack. Risk level should increase rapidly from when the predator detects the prey to when it commits to the attack. We tested this hypothesis using a predator – the echolocating bat – whose active biosonar reveals its stage of attack. We used a prey defense – clicking used for sonar jamming by the tiger moth *Bertholdia trigona*– that can be readily studied in the field and laboratory and is enacted simultaneously with evasive flight. We predicted that prey employ defenses soon after being detected and targeted, and that prey defensive thresholds discriminate between legitimate predatory threats and false threats where a nearby prey is attacked. Laboratory and field experiments using playbacks of ultrasound signals and naturally behaving bats, respectively, confirmed our predictions. Moths clicked soon after bats detected and targeted them. Also, *B. trigona* clicking thresholds closely matched predicted optimal thresholds for discriminating legitimate and false predator threats for bats using search and approach phase echolocation – the period when bats are searching for and assessing prey. To our knowledge, this is the first quantitative study to correlate the sensory stimuli that trigger defensive behaviors with measurements of signals provided by predators during natural attacks in the field. We propose theoretical models for explaining prey risk assessment depending on the availability of cues that reveal a predator's stage of attack.

## Introduction

Nearly all animals face a tradeoff between avoiding predation and seeking food and mates. This decision process is most acute when an animal detects a predator. What actions will lead to maximum fitness? What defenses should be employed and when? These questions are the subject of optimal escape theory, which holds that an animal must balance the benefits of initiating a defense (increased likelihood of survival) with the costs (e.g., expended time and energy) [1], [2].

Studies on optimal escape theory typically examine the factors that influence how close predators are allowed to approach before prey flee (flight initiation distance, FID). A greater threat to survival should lead to an increased FID, while higher costs of fleeing should decrease FID [1]–[3]). The predictions of optimal escape theory have largely been supported by empirical evidence with characteristics of the prey (e.g. morphological and behavioral defenses, amount of experience), predator (size, speed, approach trajectory), and environment (distance to refuge) consistently modulating FID [2],[4].

A critical component of escape theory is the prey's ability to recognize the threat level a predator presents, and to respond accordingly. Defensive responses are frequently graded in intensity depending on the threat level that is perceived, which has been termed “threat sensitivity” [5]. Prey frequently assign higher risk to predators that approach faster and use a more direct approach [6], [7]. A few studies have tested whether prey use other cues from the predator that could establish “intent”. Two studies on birds and lizards have found that prey use predator gaze to indicate higher risk [8], [9]; however, others found no effect of gaze on FID for deer and damselfish [7], [10].

With the mounting body of literature on optimal escape theory, efforts have recently been made to find broad rules to explain the diversity of patterns examined [3], [11]. Blumstein [11] noted a trend common to birds, mammals, lizards, and an invertebrate where FID is dependent on the distance a predator begins its approach (start distance). These results suggest that the predator's approaching movement causes the prey to flee, a finding that was not anticipated by early escape theory models [12]. Blumstein suggested a general rule that prey flee as soon as they detect a predator and identify it as a threat. He argues that this minimizes the time prey spend devoting attention to a predator. However, it was not specified how prey should determine whether a predator is a threat, and if this rule holds, how it can be reconciled with our current understanding of optimal escape theory [1], [2].

**Figure 1:**
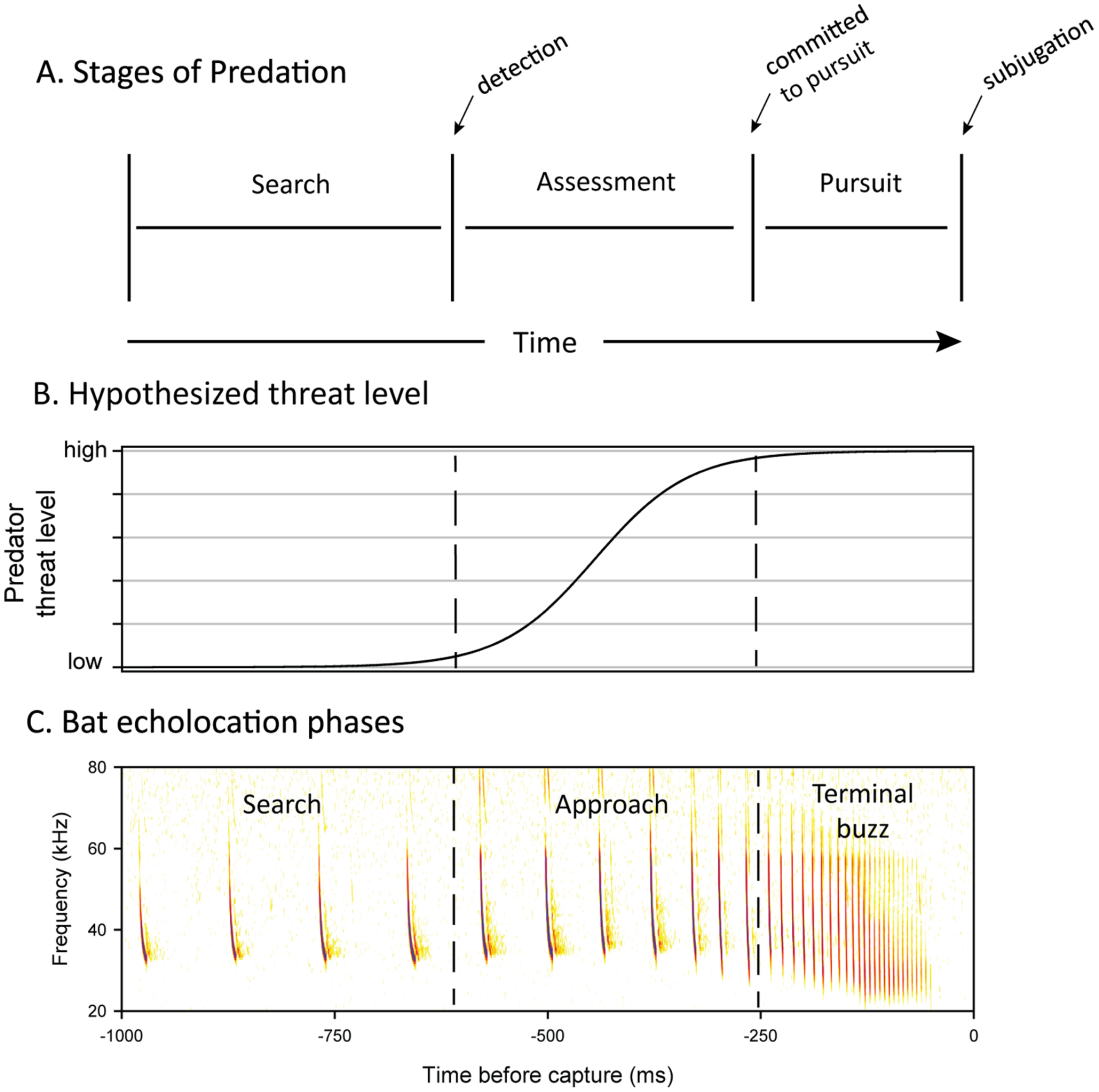
Hypothesized predator risk level as it relates to predation stage. Predation can be categorized into a series of stages (A). That presented here is modified from [13]. Predator risk is hypothesized to relate to predator stage by a sigmoidal function (B), with a rapid increase occurring from when the predator detects the prey to when it commits to the attack. (C) The bat echolocation attack sequence provides a model for studying risk relating to predator stage of attack, as the phases of bat echolocation reliably indicate the predator's attack stage. See text for explanation of specific predation stages and echolocation phases.

A successful predation sequence can be catalogued into a series of stages to aid in description and analysis [13]. A modified version of Endler's predation sequence [13] is illustrated in Figure 1A. The sequence begins with a predator searching for prey (search stage). After detection the predator will identify the prey and assess whether it is worth pursuing (assessment stage). If the predator immediately recognizes a profitable target, this stage will be rapid; however the predator may need to approach the prey to gather more information. After the predator decides the prey is profitable the pursuit stage begins. We hypothesize that predator threat markedly escalates during the assessment stage – the period from when the predator detects the prey to when it commits to the attack (Figure 1B). At this time prey defenses that prevent detection (e.g. crypsis) have failed. Unless an alternate defensive strategy is available (e.g. aposematic signaling, startle), escape is the primary remaining option. We hypothesize that prey risk assessment is driven by determining whether the predator has reached the assessment stage and how close the predator is to committing to pursuit. If evidence suggests the predator is near or at the pursuit stage, then the prey should flee, as there is little benefit to waiting. At this point, the prey's cost of attending to the predator is increasing [11] and the likelihood of escape is decreasing. While this hypothesis has been stated previously in various forms [11], [13]–[15], it has not been tested using prey responding to their naturally-behaving predators in a situation where the predator's stage of attack can be easily determined. Bats attacking insects provide just such an opportunity, as the bat echolocation attack sequence reliably indicates their stage of attack (Figure 1C).

**Figure 2:**
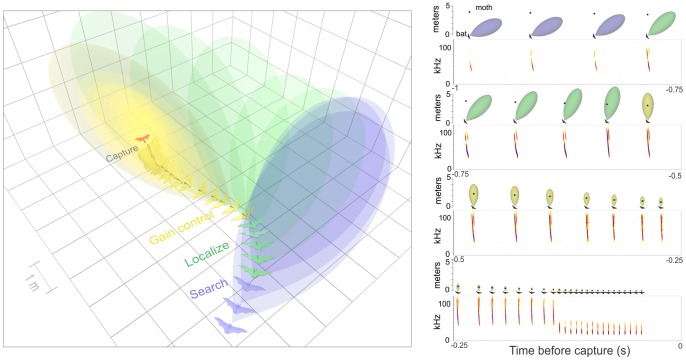
Three-dimensional simulation of the sonar beam of a bat attacking a moth (left panel), and a spectrogram of the bat echolocation sequence with two-dimensional plots of the bat's echolocation beam shape and direction relative to the target (right panel). The bat searches for prey with directional beams that are not aimed at the prey. After detection (beginning of approach phase) the bat localizes the prey and then locks its sonar beam on the target. The bat then decreases the intensity of emissions to keep echoes returning at a constant level (automatic gain control) through the late approach and buzz phases. Simulation was based on three-dimensional coordinates and bat call intensities of an attack by a *Myotis* bat. Beam shape and direction was not measured directly but was based on previous literature [19], [51]. Sonar beam shapes are depicted as the estimated volume ensonified by at least 90 dB SPL.

Bats search for insect prey by emitting echolocation (or sonar) pulses at a relatively low rate of 7–12 calls per second [16], [17]; this is termed search phase echolocation. Upon detecting a prey echo, the bat gradually increases the rate of emissions, beginning approach phase echolocation (Figure 1C). The increased emission rate allows rapid updating of the bat's “acoustic image” of the environment [18]. During the approach phase the bat localizes the target, eventually locking its directional sonar-beam to within 3° of the prey item (Figure 2) [19]. Late in the approach phase (a period sometimes referred to as the tracking phase [20]), the bat decreases the intensity of its emissions as part of a system that keeps the intensity of returning echoes at a constant level, despite decreasing target distance (automatic gain control; Figure 2) [21]–[24]. This presumably allows the bat to extract precise distance information from target echoes [20]. During this period, bats identify the target and decide whether to continue pursuit or abandon the attack [20]. Therefore, the approach phase of bat echolocation (Figure 1C) can be likened to the assessment stage of our generalized predation model (Figure 1A). In the final echolocation phase, the terminal buzz, the bat emits echolocation signals at a maximal rate (e.g. ∼160 calls/s in many species) as it attempts to intercept the prey and coordinate its final capture maneuver [25]. The terminal buzz phase can consequently be likened to the pursuit stage of our predation model (Figure 1).

Insects from at least seven orders detect bats by hearing their high-frequency echolocation calls [26]. Moths have been the focus of much research on bat-insect interactions [27]; and they have a two-part defensive response that is consistent with the threat sensitivity hypothesis [28]. Relatively quiet echolocation calls indicate a distant bat that has not detected the moth [28], [29]; they alert moths to fly away from the sound source to decrease the probability of being detected. Higher-intensity calls indicate a bat that has approached more closely, and they elicit passive or active power dives or spirals to the ground [28]. Tiger moths (Lepidoptera: Erebidae, Arctiinae; formerly Lepidoptera: Arctiidae) also respond to high-intensity bat calls with ultrasonic clicks. Depending on the chemistry and amount of sound produced by a tiger moth species, these clicks warn bats that the prey is toxic (acoustic aposematism [30], [31]) or disrupt the bat's processing of prey echoes (sonar jamming; [27], [32], [33]). The tiger moth clicking response provides a valuable model for studying prey defenses, as it can be recorded in the field under natural conditions [34] and in the laboratory in tethered animals ensonified with ultrasonic signals [35]–[38]. Because clicking is paired with diving in the sonar-jamming moth *Bertholdia trigona* [34], the initiation of clicking can be used as an indicator of when the moth begins its escape from attacking bats.

The dogbane tiger moth *Cycnia tenera* has been a model for the study of moth clicking for many decades [37]–[39]. It detects attacking bats using the acoustic cues of call intensity and call repetition rate (or its inverse, call pulse interval) [37], [40]. *C. tenera* is most sensitive to calling rates that bats use in the approach phase of attack. This is also when most tiger moths click in response to bats [35]. Why tiger moths click when they do is a matter of debate. Some have argued that clicks presented late in the attack are optimized for jamming [42] or enhancing their aposematic defense [35], [37], [43], [44]. Clicking too early could alert nearby predators to the moth's presence, or alternatively, bats may not be able to hear moths clicking at a distance [35]. We believe that optimal escape theory provides a useful framework for testing hypotheses regarding the timing of moth clicking, as this timing is under many of the same influences as the initiation of prey escape behaviors. If clicking does not conform to the predictions of escape theory, then the results could be taken as evidence that other factors (e.g. maximizing defensive effectiveness) predominate.

We studied the cues that stimulate a clicking response in *B. trigona* to test our hypothesis that prey assess predator risk by determining the stage of the predator's attack. We predicted that *B. trigona* click in response to predator cues that indicate a bat has detected and targeted them for attack. We tested this prediction by 1) determining the pulse interval and intensity thresholds that elicit clicking by broadcasting simulated bat calls to tethered moths in a sound chamber; 2) characterizing the sounds moths hear in the field when being attacked by bats (“true threat”) and when bats are attacking a nearby moth (“false threat”); and 3) comparing the acoustic properties and flight trajectories of bat passes that did and did not elicit a clicking response from *B. trigona* in the field.

## Materials and Methods

### Ethics statement

All research conducted on vertebrates (bats) involved observations of animals in their natural habitat. Bats were never captured or handled. Therefore, this work did not require state or federal permits. The methods of this study were approved by the Wake Forest University Institutional Animal Care and Use Committee (protocol #A12-048). Work was conducted with permission on private property.

### Research location and animals

Field and laboratory experiments were conducted during July 2010 at the Southwestern Research Station (American Museum of Natural History), 10 km southwest of Portal, AZ. Moths, including noctuid controls and experimental *B. trigona* (Grote, 1879), were collected and visually identified at black lights and mercury vapor lights set near riparian areas on the station grounds. Moths were held in 30 ml plastic vials for up to 48 hours prior to experimentation.

### Laboratory experiment setup

In our first experiment we determined the thresholds that elicited clicking in eight *B. trigona* moths for a range of pulse intervals (4–100 ms). Pulse interval and intensity have previously been shown to be the primary acoustic cues used by tiger moths to initiate their clicking response [37], [40]. We used a previously established experimental setup for broadcasting ultrasound signals to moths in a sound chamber [35], [36]. Briefly, moths were held with wings folded dorsally and clamped by a hemostat in a sound chamber (50 cm×20 cm ×20 cm) lined with sound-absorbent foam. An Avisoft scanspeak ultrasonic speaker (Avisoft Bioacoustics, Berlin, Germany) was placed 5 cm posterior to the moth's thorax and was used to broadcast ultrasonic digital audio files to the moth. The playbacks and the moth's clicking response were recorded by an Avisoft CM16/CMPA ultrasonic microphone connected to an Avisoft Ultrasound Gate 416H receiver. The microphone was placed 5 cm from the moth and directed perpendicular to its thorax. Ultrasound recordings were made on a laptop computer running Avisoft-Recorder software and sampling at 300 kHz. We calibrated the intensity of playbacks at the moth's position (5 cm in front of the speaker) using a Brüel & Kjær Type 2610 measuring amplifier with a Brüel & Kjær ¼” microphone (grid off).

Ultrasound playback files were generated in Matlab (Mathworks, Natick, MA, USA). They consisted of a series of 2 ms ultrasonic pulses (with 0.5 ms rise and fall times) separated by periods of silence, the duration of which varied depending on the playback file. Ten playback files were created with different pulse intervals: 4 ms, 7 ms, 12 ms, 20 ms, 30 ms, 45 ms, 60 ms, 80 ms, and 100 ms. Each digital file was 10 s long, and pulse intensities increased at a rate of 5 dB/sec from 70–120 dB SPL. To prevent an extended clicking response, playbacks were stopped as soon as the experimenter heard moth clicks. All playbacks used pure tones at 40 kHz. This is a typical frequency used by bats that attack *B. trigona* [34], and is near the peak auditory sensitivity of most moths [41].

For experimentation, moths were suspended in the dark sound chamber and trials were initiated two minutes after the moth ceased clicking. Playbacks were presented in a random order with two-minute silent periods between presentations. Only moths that routinely clicked to playbacks throughout the experiment were used in the final analysis. The audio recordings of moth clicking and playbacks were analyzed to determine the intensity which elicited a clicking response for each pulse interval. The time in the file of the first moth click was compared to the known intensity of the playback immediately preceding it, and this intensity was taken as the threshold for eliciting a clicking response.

### Field experiment overview

We conducted two field experiments where we characterized the bat sounds moths hear in their natural environment. In field experiment 1, using soundless noctuid control moths of a similar size to *B. trigona*, the bat sounds conveying “real” threats were compared to the bat sounds of “false” threats. A real threat was defined as an attack on the tethered noctuid moth; a false threat was an attack on a neighboring, free-flying moth. In field experiment 2, we recorded bats flying near and sometimes approaching tethered *B. trigona*. We then compared acoustic and flight parameters of bat passes that elicited a clicking response with those that did not elicit a response.

### Field experiment setup

**Figure 3:**
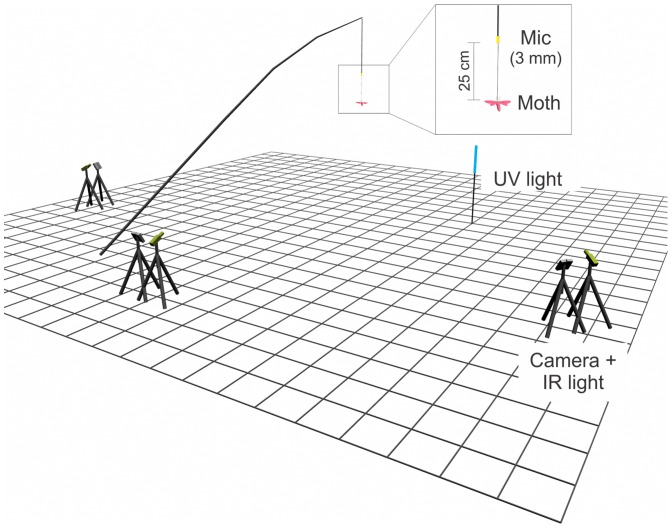
Three-dimensional schematic of field setup for recording bat attacks on tethered and free-flying moths. Three infrared cameras recorded bat flight trajectories, positions of tethered and free-flying moths, and positions and directional axes of the ultrasound microphone. An ultraviolet light attracted free-flying moths and foraging bats to the observation area. In field experiment 1 a silent noctuid moth was tethered and suspended along with a miniature (3 mm diameter) microphone from a 10 m telescoping pole. The microphone's close proximity to the tethered moth allowed for estimating bat call intensities emitted in the moth's direction (10 cm from the bat's mouth) and arriving at the moth for attacks on the tethered moth (real attacks) and attacks on nearby free-flying moths (false threats). In field experiment 2, the clicking moth *Bertholdia trigona* was tethered below the microphone and bat passes that elicited a clicking response were compared to passes that did not elicit clicking.

We recorded infrared video and ultrasound of free-flying *Myotis* bats (see below for species identification methods) attacking tethered and free-flying moths in a gap in a forested riparian area. Figure 3 illustrates the components of the field setup. Individual moths and a miniature ultrasonic microphone (Knowles Acoustics FG-3329, Itasca, IL, USA) were suspended from the end of a 10-m telescopic pole that was anchored into the ground at a 45-degree angle. This arrangement, combined with three-dimensional reconstruction of bat flight trajectories, moth positions, and microphone positions, allowed us to calculate the bat call sound pressure levels at the point of emission (source level) using an arbitrary distance of 10 cm from the bat, and at the moth's position. These calculations are described below. A 3-mm diameter shielded microphone cable ran from an AR100 ultrasound receiver (Binary Acoustics Technology, Tucson, AZ, USA) along the length of the pole and down from the pole's tip (Figure 3). Ultrasound was recorded on a laptop computer running Spect'r software (Binary Acoustics Technology) sampling at 250 kHz. Individual moths were suspended by a 25 cm monofilament line from the end of the microphone cable (Figure 3).

In preparation for tethering, we removed scales from the moth's mesoscutum and affixed to it a small (5 mm) loop of monofilament line with gel superglue (Loctite brand). At the time of experimentation, the moth was tied to the monofilament line on the telescoping pole by the affixed loop and then hoisted into the air. The pole was shaken periodically by the experimenter to add motion to the tethered moth and to keep the moth flying. A 15-watt ultraviolet light (Leptraps LLC, Georgetown, KY, USA) attached to a 1.5 m pole near the hoisted moth was used to attract insects, and therefore foraging bats, to the recording area.

### Bat species identification

Bats were identified to the genus *Myotis* based on a diagnostic feature of their search-phase calls – a terminal downward sweeping “tail” that follows a more shallowly frequency-modulated call component [45]. Species-level identification was not possible because of incomplete sampling of some species' call repertoires and the large degree of overlap that occurs in the acoustic characteristics of the six species in the genus *Myotis* (*M. auriculus*, *M. californicus*, *M. ciliolabrum*, *M. thysanodes*, *M. velifer*, and *M. volans*) present at our field site [34].

### Videography

Three infrared-sensitive Basler Scout cameras (model scA640-120 gc; Ahrensburg, Germany) recording at 60 frames-per-second at 640×480 resolution were placed around the experimental area and focused on the tethered moth. Cameras were hard-wired to start simultaneously by a signal sent from a custom Innovision Systems (Columbiaville, MI) synchronization box, which was controlled by MaxTraq software (Innovision Systems) running on a desktop computer. Infrared illumination was provided by 12 Wildlife Engineering IR-Lamp6 lights (Tucson, AZ, USA), and two Bosch UFLED20-8BD illuminators (Farmington Hills, MI, USA).

### 3-D calibration and reconstruction

Using the relative orientation method [46] in MaxTraq3D (“Dynamic wand method”; Innovision Systems) and the video recordings by the three cameras, the three-dimensional positions of bats, moths, and the microphone were reconstructed. This approach has been used recently for studying bat-moth interactions in the field and is explained in more detail elsewhere [34]. The accuracy of our reconstructions was tested by moving a “wand” (two spherical infrared markers fastened to a rod at fixed distance from each other) throughout our calibrated volume and comparing the known distance between the two markers to that measured using our 3-D reconstructions. Over 1500 frames, we found a mean error of 0.4 cm for the two markers set 145 cm apart (0.28% error). Our calibration volume was approximately 6 m (width) by 6 m (depth) by 4 m (height) or 144 m^3^.

Two infrared tape markers were placed at known positions on the microphone cable in order to determine the microphone's position and directional axis. The “center-of-mass” of the bats, moths and two microphone markers were digitized from our video using MaxTraq2D (Innovision Systems). These 2-D data were transformed into 3-D coordinates using MaxTraq3D. The 3-D coordinates were then imported into Matlab, where a custom program (BATracker.m, coded by B. Chadwell and A. Corcoran) was used to fit a quintic smoothing spline to the 3-D data. This spline function was used to generate bat flight vectors, bat-moth vectors, bat approach angles, and other flight parameters [33], [34], [47].

### Audio calibration

We used our audio recordings and 3-D reconstructions to determine the SPLs of bat calls at the tethered moth's position and the source level at an arbitrary distance of 10 cm from the bat [dB peak equivalent SPL (peSPL) re. 20 µPa] [48]. Bats emit directional calls [19], [49]. The estimated SPLs for bat emissions represent that emitted in the moth's direction. Early in the attack, until the bat has locked its sonar beam on the target, the moth is typically off-axis from the bat's sonar beam emission (Figure 2) [19], and estimated emission SPLs are likely less than the maximum emitted by the bat.

Bat call intensities were first determined at the position of the microphone using several adjustment factors. The frequency response of the Knowles microphone was determined by playing pure tone signals from an Avisoft ScanSpeak Ultrasonic speaker (Avisoft Bioacoustics, Berlin, Germany) to the exact microphone, cable, and receiving unit used in the experiments. Audio was recorded at the same position (50 cm directly in front of the speaker) by our Knowles microphone and by a Brüel & Kjær ¼” microphone (grid off) connected to a Brüel & Kjær Type 2610 measuring amplifier. Playbacks were delivered at 5 kHz increments from 20–100 kHz. These values provided a frequency-specific conversion from recorded voltage to SPL. The frequency response was fairly flat (±1.5 dB) from 20–40 kHz, followed by decreased sensitivity of approximately 5 dB per 10 kHz from 40–100 kHz. This response resulted partly from the long, thin microphone cable which was important for our experimental design. For 20 kHz, 30 kHz, 40 kHz, 50 kHz, and 60 kHz (the range of potential peak frequencies used by bats in this study) the directionality of the Knowles microphone was determined in 22.5° increments from the microphone pointing directly at the speaker to directly away from the speaker. The microphone was fairly omnidirectional with adjustments ranging from ± 2–4 dB depending on frequency and angle.

Attenuation due to spherical spreading and excess atmospheric attenuation were accounted for using the temperature and humidity recorded at the beginning of each session [50]. There may have been a difference in the recorded SPL at the microphone's position and the moth's position due to the directionality of the bat call. This was minimized by having the microphone as close to the moth as possible while still allowing bats to interact naturally with the tethered prey. To account for this we measured the angle between the bat-moth vector and the bat-microphone vector throughout each attack. *Myotis* bat call directionality depends on the phase of attack, with search and approach phase calls having −6 dB horizontal beam width of 80° (40° off axis in either direction) and buzz phase calls having -6 dB horizontal beam width of 180° (90° off axis) [51]. During search and approach phase the angle measured between bat-moth vector and bat-microphone vector was 23.5 ± 9.7° and for buzz phase the angle was 44.4± 17.3°. To compensate for this, we added 3 dB to our calculations of bat call SPLs; ± 1–3 dB of error in our calculations cannot be accounted for since the exact direction of bat calls was unknown.

For our second field experiment involving tethered *B. trigona*, we compared pulse intervals and intensities of bat passes that elicited clicking to those that did not elicit clicking. In attack sequences in which the moths clicked, signal intensity was determined by averaging the intensity of the two bat calls preceding the clicking response. Pulse interval was measured as the time between the beginnings of these two calls. In attack sequences in which the moth did not click, intensity was measured as the average of the most intense call, the call before, and the call after. Pulse interval was measured as the average of the time between these three calls.

### Statistical analysis

For our laboratory experiment, repeated-measures Analysis of Variance (ANOVA) was used to determine whether moths have different clicking intensity thresholds for pulse intervals representing different phases of echolocation attack. Thresholds were averaged for representative pulse intervals for each phase (search: 80 and 100 ms; approach: 20, 30, and 45 ms; and buzz: 4 and 7 ms). Thresholds for pulse intervals of 60 and 12 ms were not used as they are transitional between phases. Planned Bonferroni pairwise comparisons were used to determine whether means were significantly different [52].

We determined the relationship between time and call intensity from our first field experiment for three conditions: real threat aligned by the end of the attack, real threat aligned by the bat call of maximum intensity, and false threat aligned by the end of the attack. These data had a nested structure, with many calls coming from each bat attack. To account for this we used linear mixed-effects models with “bat attack” as a random effect, time, and time squared as fixed effects (to determine if the relationship was linear or quadratic), and call intensity as the response variable. This was repeated once for source level intensity and once for intensity at the moth's position. We used ANOVA to test whether models with random effects better fit our data than models without random effects. Significance of each fixed effect variable was first determined from a single model including all variables (using α = 0.05). Non-significant variables were then removed, and the final model using only significant random and fixed effects was computed. We closely followed the protocol recommended by Zuur et al. [53]. Analyses were conducted in the statistical program R using the “lme” command from the “nlme” package [54], [55].

Discriminant Function Analysis (DFA) was used to determine an optimal threshold for differentiating real and false threats based on pulse interval and intensity. DFA was also used to determine what acoustic and flight variables differ between bat passes that elicited and did not elicit clicking by *B. trigona* in the field. Stepwise forward variable selection was used with a P-to-enter of 0.05 [56]. A Receiver Operating Characteristic (ROC) curve was used to determine the degree of overlap in call intensities between real and false threats for each echolocation phase. The area under the ROC curve (AUR), which equals the likelihood a randomly chosen call from the real threat group has a higher intensity than a randomly chosen call from the false threat group, was used as our measure of the degree of overlap [57].

Finally, to help determine what cues may be stimulating *B. trigona* to click in the field, we conducted linear mixed-effects models to establish the relationship between (1) time and bat call intensity and (2) time and pulse interval for a 250 ms period prior to when moths clicked. For this analysis we used the same protocol described above for analyzing call intensity data for real and false threats.

## Results

### *Bertholdia trigona* clicking thresholds

**Figure 4:**
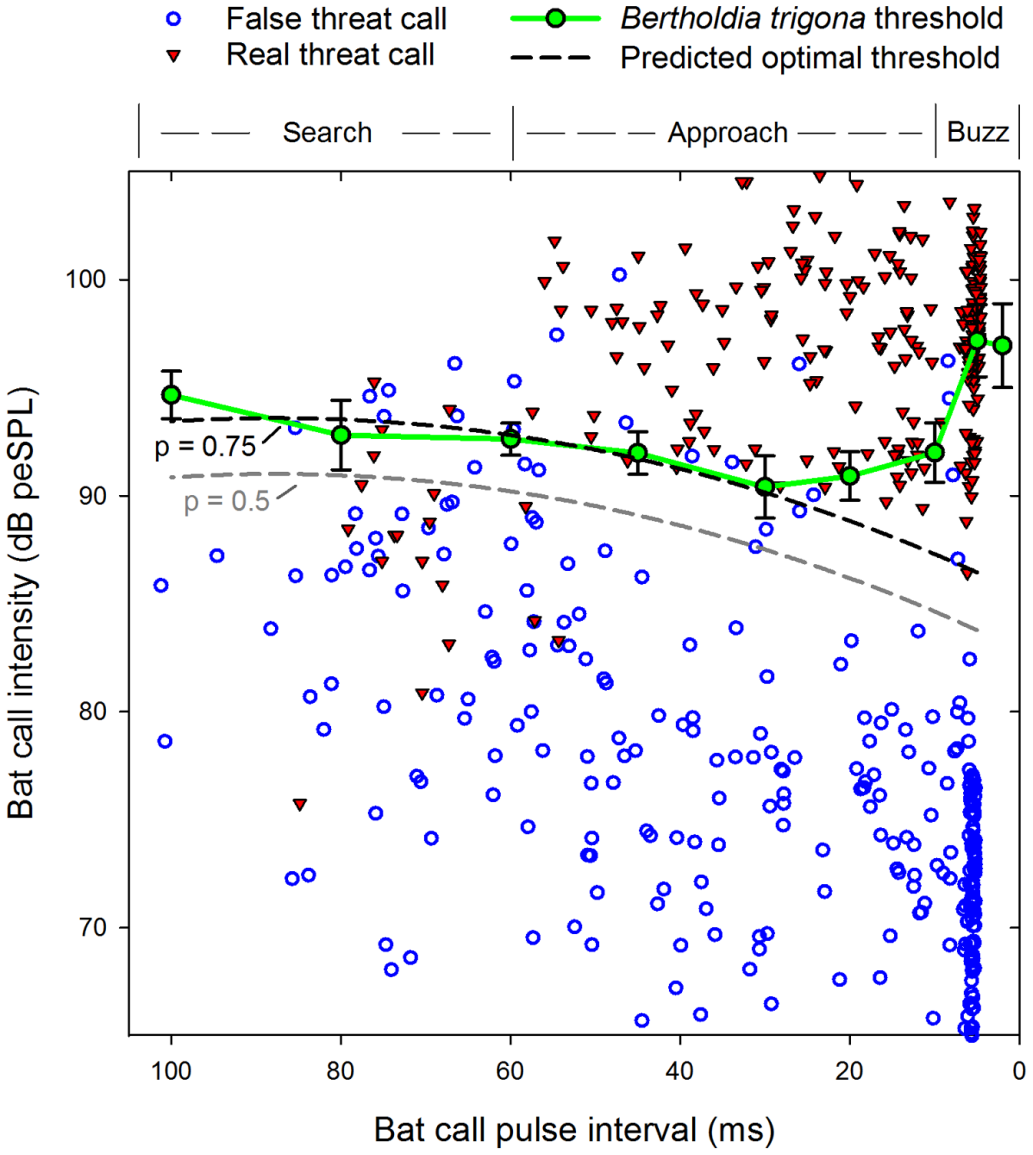
Predicted and measured clicking thresholds for different bat echolocation pulse-intervals in *Bertholdia trigona*. Echolocation call intensity and pulse interval impinging upon a tethered focal moth are shown for five attacks on individual noctuid moths (real threat) and nine attacks on nearby, free-flying moths (false threat; see figure 2). The optimal threshold for discriminating real and false threats was determined using quadratic discriminant analysis with 50% (p = 0.5) and 75% (p = 0.75) likelihood of a call being assigned as a real threat. *Bertholdia trigona* clicking thresholds (mean ± s. e.) measured from eight moths suspended in a sound chamber matched predicted p = 0.75 thresholds for pulse intervals of 35–100 ms.

From our laboratory experiment, we found that *B. trigona* had the lowest clicking thresholds for pulse intervals in the late approach phase of bat echolocation (20–30 ms pulse intervals; green symbols in Figure 4). Intensity thresholds were significantly different between echolocation phases (Repeated measures ANOVA; N = 8; d.f. = 7, 2; F = 19.2; P<0.0001); thresholds for search (P = 0.015) and terminal phase (P = 0.001) pulse intervals were higher than those for approach phase pulse intervals.

### Discriminating real and false threats

To determine whether it is possible for a moth to differentiate real and false threats, bat call pulse intervals and intensities (the cues used by tiger moths for identifying an attack) for five attacks on five tethered, soundless noctuid moths (real threat) and nine attacks on nearby soundless moths (false threat) were plotted (Figure 4). We compared the degree of overlap in call intensities between real and false threats for each echolocation phase using the area under the ROC curve (AUR), which equals the likelihood a randomly chosen call from the real threat group has a higher intensity than a randomly chosen call from the false threat group. Search phase calls had a moderate degree of overlap between the two conditions (AUR = 0.72; P = 0.009); however, approach (AUR = 0.97; P < 0.0001) and terminal phase calls (AUR = 0.997; P < 0.0001) had a large degree of separation. Using quadratic discriminant analysis we determined the optimal thresholds for discriminating real and false threats with 50% (p = 0.5) and 75% (p = 0.75) likelihoods of the threat being real. Measured *B. trigona* clicking thresholds matched predicted p = 0.75 thresholds for pulse intervals of 35–100 ms, but not for pulse intervals at or below 20 ms (Figure 4). Pulse intervals in the terminal buzz had the worst fit. These results suggest that *B. trigona* clicking thresholds are optimized for differentiating predator threat levels for bats in search phase and most of approach phase, but not for bats in the terminal buzz.

### Characterization of real and false predator threats

**Figure 5:**
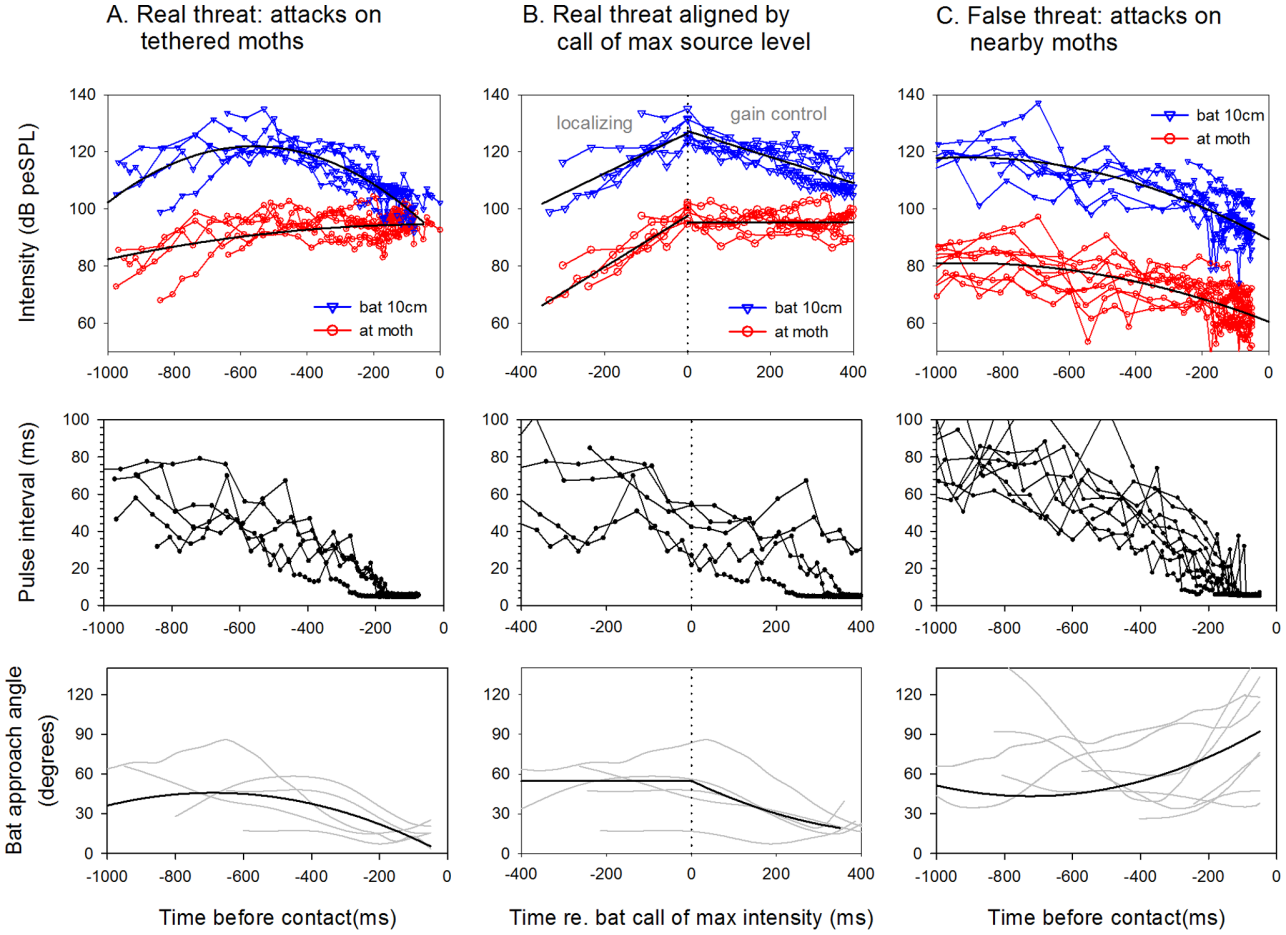
Characterization of “real” and “false” threats. A real threat is a bat attacking the target moth (A, B); a false threat is a bat attacking a nearby moth (C). Real threats are shown (A) aligned by the end of the attack, and (B) aligned by the echolocation call of maximum intensity. The top panels show the bat echolocation intensities 10 cm from the bat (emitted in the moth's direction) and at the moth's position. Middle and bottom panels show how bat pulse intervals and approach angles change over the course of an attack, respectively. The rise in bat call intensity early in attacks in A and B reflects the bat localizing the moth, followed by an immediate decrease in emission intensity, which contributes to the bat's automatic gain control (see text and Figure 2) [21]. Bats engage gain control (time = 0 s in B) in the middle of approach phase and then soon turn towards the prey (a decrease in approach angle). False threats are characterized by decreasing call intensities at the moth's position and approach angles that are poorly correlated with time in the attack (see Table 1 for statistics).

To understand further the cues available to moths for differentiating predator threat levels, we characterized the bat sounds arriving at a moth when it was being attacked (real threat) and when a nearby moth was being attacked (false threat). All sound levels were indicators of the intensity being directed towards the moth, which was not always equal to the maximum intensity emitted by the bat. We first report the results of the real threat scenario with bat calls aligned by the end of each attack (Figure 5A). Source levels gradually increased as the bat directed its beam towards the prey, followed by a decrease as gain control was engaged; call intensities at the moth's position gradually increased until nearly plateauing near the end of attacks (Table 1). As expected, bat approach angles decreased sharply near the end of attacks (Figure 5A; Table 1).

**Table 1:** *Myotis* call intensities and approach angles with respect to time for real and false attacks.

		N_attacks_	N_calls_	B_time_ *	B_time_^2^ *	P_time_ †	P_time_^2^ †	P_attack_ †	R^2^
**Real threat**,	Source level	5	220	114.0	–102.1	**<0.0001**	**<0.0001**	**<0.0001**	**0.62**
**time to end of**	Intensity at moth	5	220	–	−12.3	0.15	**0.003**	**<0.0001**	**0.28**
**attack**	Approach angle	5	–	136.4	−99.1	**<0.0001**	**<0.0001**	**<0.0001**	**0.35**
**Real threat**,	Source level	5	31	70.4	–	**<0.0001**	0.87	**<0.0001**	**0.70**
**calls before max**	Intensity at moth	5	31	89.6	–	**<0.0001**	0.76	**0.003**	**0.84**
**source level**	Approach angle	5	–	–	–	0.09	0.57	**<0.0001**	0.01
**Real threat**,	Source level	5	189	−45.5	–	**<0.0001**	0.32	**<0.0001**	**0.63**
**calls after max**	Intensity at moth	5	189	–	–	0.16	0.96	**<0.0001**	0.13
**source level**	Approach angle	5	–	152.1	144.7	**<0.0001**	**<0.0001**	**<0.0001**	**0.38**
**False threat**,	Source level	9	277	63.2	−34.8	**<0.0001**	**<0.0001**	**<0.0001**	**0.37**
**time to end**	Intensity at moth	9	277	44.9	−24.5	**<0.0001**	**<0.0001**	**<0.0001**	**0.34**
**of attack**	Approach angle	9	–	160.9	108.0	**<0.0001**	**<0.0001**	**<0.0001**	**0.10**

* Units of B_time_ are dB•s^−1^ for source level and intensity at moth and degrees•s^−1^ for approach angle. Units of B_time_^2^ are dB•s^−2^ for source level and intensity at moth and degrees•s^−2^ for approach angle.

† The variables time and time^2^ were added as fixed effects. Attack number was entered as a random effect.

We next analyzed call intensities from the real threat scenario with calls aligned by the call of maximum intensity (Figure 5B). This call represents the time bats switch from localizing prey to gain control (Figure 2). Source levels increased linearly during localization and then decreased linearly during gain control (Table 1). At the moth's position bat call intensity increased linearly during localization. During gain control call intensity at the moth's position was statistically constant with a mean of 95.3 dB (Table 1). Bat approach angle was independent of time during localization and decreased during gain control (Figure 5B).

In contrast to the real threat scenario, in the false threat scenario bat call source levels and intensities at the moth's position decreased as attacks progressed (Figure 5C; Table 1). The bat's flight direction relative to the tethered, non-target moth was largely independent of the time from the end of the attack (indicated by low R^2^; Table 1), with the exception that bats tended to veer away from the non-target moth during the last 200–300 ms of the attack.

### Clicking responses of tethered moths in the field

**Figure 6:**
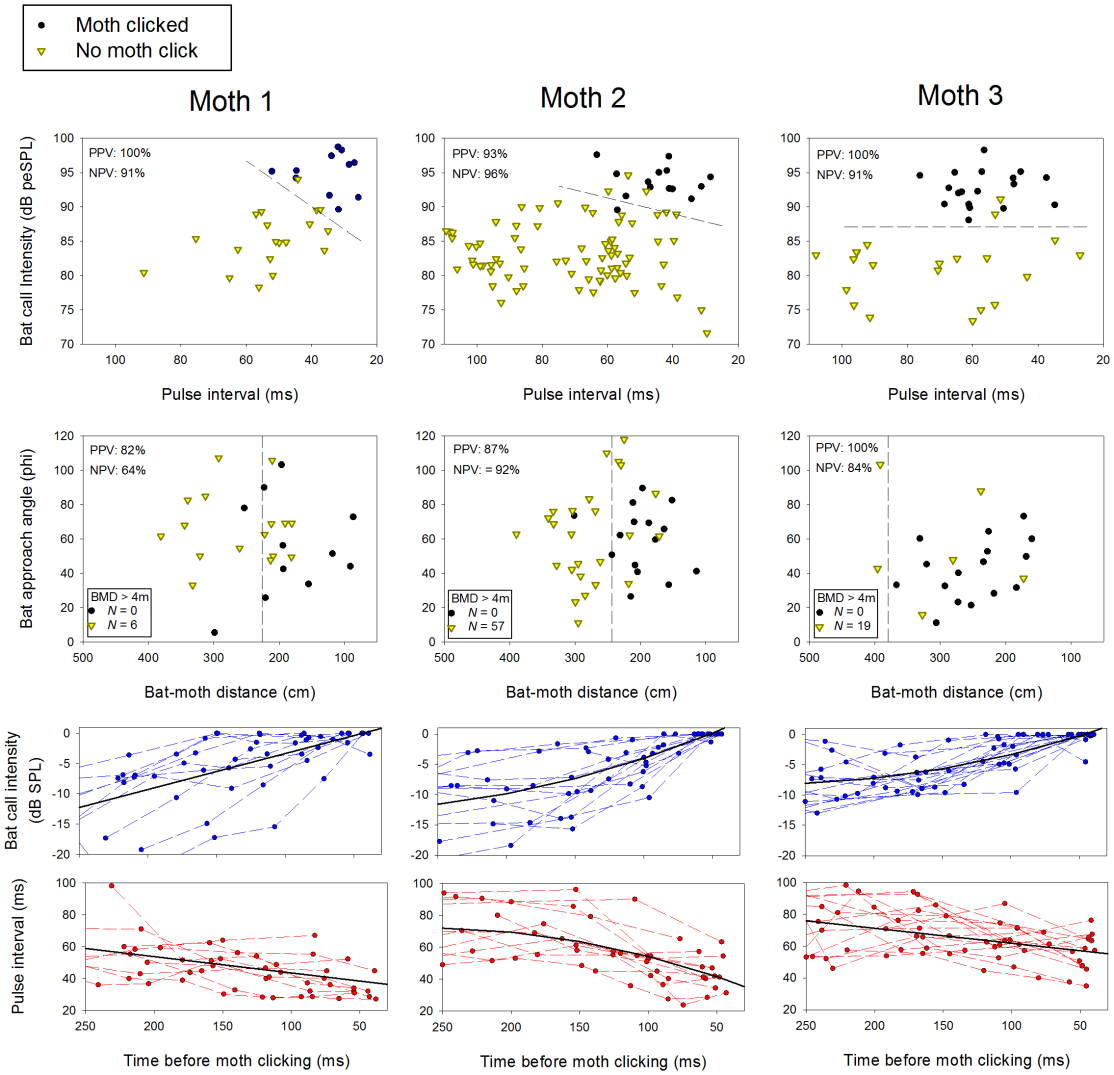
Discrimination of bat passes that did or did not elicit clicking by tethered *B.* *trigona* in the field. Acoustic characteristics (bat pulse interval *vs*. bat call intensity) and bat flight characteristics (distance from moth *vs*. flight trajectory relative to bat-moth vector, or phi) were used to differentiate bat passes that elicited a clicking response and those that did not (Top two rows of graphs). Dashed lines indicate thresholds for discriminating clicking from non-clicking interactions using discriminant function analysis. PPV, positive predictive value, or the percent of clicking interactions correctly classified; NPV, negative predictive value, or the percent of non-clicking interactions classified correctly. Some interactions were included where bats were outside the calibrated volume of the cameras and bat-moth distance (BMD) was>4 m. Exact BMD and bat approach angles were not available for these interactions. Bat call intensities increased and pulse intervals decreased prior to moth clicking (bottom two rows of graphs; see Table 2 for statistics).

Using tethered *B. trigona* in the vicinity of free-flying bats, we determined how well two groups of characteristics – bat call properties and bat flight trajectories – could discriminate interactions where moths did or did not click. This analysis used 11–16 bat passes that elicited clicking and 19–83 passes that did not elicit clicking per moth for three moths. Bat call intensity at the moth's position was significant in predicting whether or not moths clicked for all three moths tested (DFA; P<0.0001); pulse interval was a significant discriminating variable for two of three moths tested (DFA; P<0.0001), with shorter pulse intervals (higher repetition rates) being more likely to elicit a clicking response (Figure 6). Moths typically clicked in response to bats in early to middle approach-phase (25–60 ms pulse intervals). Moth 3 also responded to bats in late search-phase (60–70 ms pulse intervals). Moth clicking was preceded by bat calls that increased in intensity and slightly decreased in pulse interval (bottom two rows of Figure 6; Table 2), both indications that the moth had recently been detected and targeted by the bat.

**Table 2:** *Myotis* call intensities and pulse intervals with respect to time before clicking for three *Bertholdia trigona* moths.

		N_attacks_	N_calls_	B_time_ *	B_time_^2^ *	P_time_ †	P_time_^2^ †	P_attack_ †	R^2^
**Moth 1**	Intensity at moth	11	59	59.5	–	**<0.0001**	0.91	**<0.0001**	**0.45**
	Pulse interval	11	59	−102.8	–	**0.008**	0.42	**<0.0001**	**0.21**
**Moth 2**	Intensity at moth	14	64	106.9	−161	**<0.0001**	**0.0011**	**0.0002**	**0.48**
	Pulse interval	14	64	−353.9	673.6	**<0.0001**	**0.0013**	**0.0002**	**0.28**
**Moth 3**	Intensity at moth	16	71	84.3	−150.4	**<0.0001**	**0.0045**	0.12	**0.45**
	Pulse interval	16	71	−94.0	–	**0.0003**	0.65	0.99	**0.17**

* Units of B_time_ are dB•s^−1^ for source level and intensity at moth and degrees•s^−1^ for approach angle. Units of B_time_^2^ are dB•s^−2^ for source level and intensity at moth and degrees•s^−2^ for approach angle.

† The variables time and time^2^ were added as fixed effects. Attack number was entered as a random effect.

For interactions where bats flew through the calibrated volume, bat distance (DFA; P<0.01), but not approach angle (P>0.1), significantly discriminated between clicking and non-clicking cases for moths 1 and 2 (Figure 6). Neither variable was significant for moth 3 (DFA; P>0.1). All bat passes outside of the calibrated volume (which had a minimum distance of 4 m from the moth) failed to elicit a clicking response. Because a threshold could not be determined for moth 3 using only passes occurring within the calibrated volume, we conducted an ROC analysis on bat distance using interactions in and out of the calibrated volume. This analysis revealed a statistically significant threshold (Figure 6, moth 3; P<0.001). Moths 1 and 2 had similar bat distance clicking thresholds, which were about 1.5 m less than the threshold for moth 3. Acoustic characteristics were better at predicting whether moths clicked than bat flight characteristics. Acoustic characteristics correctly predicted an average of 98% of interactions where moths clicked (positive predictive value; PPV) and 93% of interactions where moths did not click (negative predictive value; NPV). Flight characteristics had an average PPV of 90% and an average NPV of 80%. This indicates that there were several cases where a bat's flight trajectory could not explain the moth clicking response, but bat acoustic characteristics could.

## Discussion

Using a combination of field and laboratory experiments, we tested the hypothesis that moths initiate defensive clicking in response to acoustic characteristics that reveal they have been detected and targeted by a bat. First, we conducted a laboratory experiment to determine moth clicking thresholds for a range of pulse intervals. As has been found previously for the dogbane tiger moth *Cycnia tenera* ([37], [40]), *B. trigona* had the lowest thresholds in the late approach phase (20–30 ms pulse intervals) (Figure 4, green lines). In an attack sequence, a bat produces these sounds just before committing to the attack, a time when predator threat level was predicted to be nearing its maximum (figure 1).

How do moth thresholds compare to the sounds moths are exposed to under natural conditions? To answer this question we compared the sounds moths hear when they are getting attacked (real threat) to those heard when a nearby moth is being attacked (false threat). We found that *B. trigona* clicking thresholds closely matched predicted optimal thresholds for discriminating real and false threats for search and most of approach phase echolocation (Figure 4). These are the phases for which it is most important for a moth to determine whether it is under attack. After this point, in the terminal buzz, it may be too late for clicking to be effective [33]. Moths may also have difficulty accurately encoding buzz phase echolocation in their nervous signals [40], and moths may not be able to discriminate buzz phase echolocation from insect stridulations [37], however this has not been tested empirically.

By looking at the degree of overlap in call intensities of real and false threats at different pulse intervals (Figure 4), it is clear that search-phase calls are ill-suited for discriminating predator threat levels. Early approach phase (30–60 ms pulse intervals) is the first time in an attack that cues are available to distinguish real and false threats, and this is when tethered (Figure 6) and free-flying moths [34] click in response to naturally foraging bats. This is also when we predicted predator threat level would begin to rise sharply (Figure 1).

In early approach phase, the bat is directing its sonar beam increasingly towards its prey as it begins to decrease its pulse interval (Figure 2). The prey perceives this as a rapid increase in intensity and a small decrease in pulse interval (time -200 ms to 0 ms in Figure 5B). These cues are present even before the bat begins turning toward the moth. This potentially provides a substantial advantage for the prey. A neighboring moth that is not being targeted hears a decrease in pulse interval, however it does not hear a rise in intensity because the bat has not directed its sonar beam its way (Figure 5C). This neighboring moth will soon hear the bat decreasing its intensity as it engages automatic gain control, a cue that could further indicate the moth is not being targeted.

Together, these data support the hypothesis that *B. trigona* clicking thresholds have been shaped by natural selection to discriminate between legitimate and false predatory threats. Moths appear to initiate clicking at the earliest time when they can determine that they have been detected and targeted by an echolocating bat. In the case of sonar jamming, it is not necessary to include other explanations for the timing of moth clicking, for example that moths click at a particular time in an attack when jamming is most effective [35], [42]. *Bertholdia trigona* moths click well before bats have fully committed to an attack (which occurs after bats gain more information about prey in late approach). However this may be expected because bats that have detected moths the size of *B. trigona* nearly always commit to pursuit unless a defense such as sonar jamming is presented (Corcoran and Conner, 2012). Being attacked is a near certainty for a moth that has been detected and localized by a bat.

How do these results contribute to our understanding of optimal escape theory? We hypothesized that prey assess predator risk based on where the predator is in its attack sequence, with risk increasing after detection and as the predator progresses toward committing to the attack (Figure 1). Our results support this hypothesis, as moths reliably began their jamming defense in response to cues that indicated they had recently been detected and targeted. This happened when bats were at distances of 1–3 m (Figure 6), just inside the range where bats detect moths (2.2–4.5 m) [34]. Moths did not click in response to several bats that flew within the distance expected to excite a response (Figure 6). Acoustic features of bat calls were highly accurate at predicting whether moths clicked (Approx. 95%), and even more so than bat flight characteristics. Therefore it appears that these bats did not direct their sonar beams at the moths, and the moths correctly ignored false threats.

Growing evidence suggests that prey dynamically respond to predator behaviors that indicate the predator's stage of attack. Birds, mammals, lizards and invertebrates have been shown to wait a fixed amount of time after a predator begins approaching to initiate escape, regardless of the predator's distance [7], [11], [12], [15], [58], [59]. This response is more common when predators move rapidly, supporting the idea that prey perceive these movements as a directed attack [15], [59]. Prey also flee from predators (usually humans) at greater distances when they approach more directly [6], [7], turn towards prey [60], [61], move faster [7], [62], or approach while gazing at the prey [8], [9]. However, these finding are not universal. For example, deer do not appear to respond to predator gaze or the presence of a gun [7]) and lizards do not flee from an approaching distant predator if it moves slowly [15], [59]. Different prey, therefore, use different predator cues to assess risk. This may be a result of differing sensory capabilities, predators that provide different cues in their approach, or differing environmental conditions [63]. Further comparative work is needed, especially with studies of naturally behaving predators and prey where predator cues, and prey responses to them, can be measured.

Bats and moths provide a useful system for testing the hypothesis that prey determine predator risk based on the predator's stage of attack because bats inadvertently advertise when they have detected the prey and are committing to an attack. For many predator-prey systems this is unlikely to be the case, and there may be selective pressure for predators to conceal their stage of attack. For example, some bats use “stealth echolocation” – that is, they echolocate quietly, which prevents moths and other eared insects from detecting an attack [29]. Depending on the animals involved and environmental conditions, prey will have differing levels of information about a predator's stage of attack.

**Figure 7:**
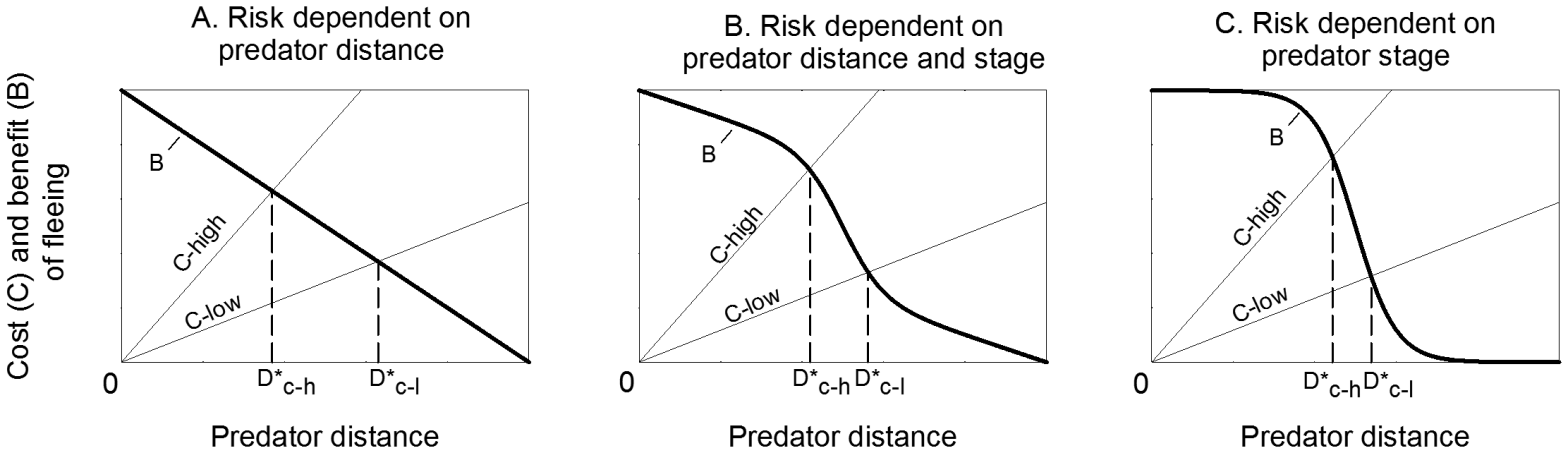
Cost-benefit models of escape behavior with varying prey knowledge of predator attack stage. Prey cost of fleeing C is dependent on predator distance and prey characteristics (C-low vs. C-high). In the classic model (panel A), benefit of fleeing B is equal to predator risk and increases as the predator gets closer (modified from [1]). The optimal escape distance D* occurs at the intersection of B and C. We propose that this model holds for prey that lack information about predator attack stage. For prey with information about predator attack stage (panel C), B is predicted to be a sigmoidal function with an increase in risk/benefit of fleeing when the predator detects a target and commits to an attack (see Figure 1). Partial information on predator stage has a risk function that is a combination of predator distance and attack stage (panel B). Increased availability of information on predator stage diminishes the effect of prey cost level (C-high vs. C-low) on optimal escape distance D*.

We propose that a prey's ability to perceive cues that reveal the predator's stage of attack alters the shape of the predator risk assessment curve (Figure 7). Predator risk (which is equal to the benefit of fleeing) is typically presented as a linear [2] or quadratic [1] function of predator distance, with risk increasing as the predator approaches. The cost of prey fleeing is assumed to increase with predator distance (Figure 7A) [1]–[3]. The intercepts and slopes of these functions can change depending on factors relating to the prey, predator, and environment [3].These factors shift the intersection of the prey cost and benefit functions, and therefore the distance where it is optimal for prey to flee (D*). We argue that this model holds for prey that have little or no information about predator attack stage. For prey that have more complete information on predator attack stage (such as in the current study), we propose that the predator risk function is sigmoidal and risk increases rapidly in the predator's assessment stage (Figure 1). The predator's distance where it detects and assesses prey would differ between interactions, and therefore the predicted FID would vary depending on the predator's detection/assessment distance. The sigmoidal shape of the risk function reduces the effect that prey flight costs have on the optimal FID (Figure 7C). A prey's cost of fleeing is rarely if ever greater than the threat posed by a lethal predator intent on capturing it. There may also be cases where prey have partial information on predator attack stage. In this case we suggest that the predator risk function would be a combination of the linear and sigmoidal functions relating to predator distance and attack stage (Figure 7B). This function may also relate to situations where prey have information on predator attack stage but are at a disadvantage if they are discovered at close range. This mixed model is similar to a model where risk shifts from a lower to a higher risk function when prey recognize a change in predator behavior [59].

The proposed models offer a theoretical explanation for the previous assertion that prey should flee as soon as they detect a predator and determine it is a threat [11]. When prey can easily determine a predator's attack stage (such as with moths and bats), that factor overwhelms decision making. Prey costs and predator distance become less important. When it is unknown whether a predator is attacking, prey must use indicators like predator distance and approach speed to estimate the likelihood of attack. In this case risk level may rise more slowly as the predator approaches, and prey costs will have a greater effect on FID.

In summary, we have provided some of the first quantitative evidence linking prey defensive thresholds with cues measured from natural predatory attacks. *Bertholdia trigona* use relatively simple rules for determining when to initiate clicking based on two acoustic parameters: pulse interval and intensity. These rules, which are encoded in the moth's nervous system (see discussion in [37]), allow moths to respond rapidly soon after being targeted by echolocating bats. They also allow moths to discriminate between legitimate and false predatory threats with remarkable accuracy. This ability is facilitated by a predator who nearly continuously advertises its stage of attack through echolocation. These findings demonstrate that economic considerations determine the time at which *B. trigona* exhibits its combined jamming-and-diving defense, despite the possibility that other factors such as timing the defense for maximum effect [42] may be providing conflicting selective forces.
